# Supplementary material for: TDP-43 and Alzheimer’s Disease Pathology in the Brain of a Harbor Porpoise Exposed to the Cyanobacterial Toxin BMAA
Source: Toxins (Basel). 2024 Jan 12;16(1):42. doi: 10.3390/toxins16010042 (PMC10821503; doi:10.3390/toxins16010042)
Supplement: Supplementary file 1 [file toxins-16-00042-s001.zip › toxins-2772882-supplementary.pdf]

TDP-43 and Alzheimer's Disease Pathology in the Brain of a Harbor Porpoise Exposed to the Cyanobacterial Toxin BMAA

Table S1. Primer designs based on *T. truncatus* and *D. delphis* sequence.

Gene Symbol	Gene Name	Assay ID	Assay Name	SeqAC	Transcript variant	Amplicon Length	Location
APP	Amyloid β precursor protein	APGZHYX	APP_1	XM_019922563.1	X1	64	550
		APGZHYX	APP_1	XM_019922564.1	X2	64	550
C9orf72	Chromosome 9 open reading frame 72	APKA64T	C9ORF72_1	XM_004318999.2		61	817
GRN	Granulin precursor	APRWHFG	GRN_1	XM_019939325.1		64	158
MAPT	Microtubule associated protein tau	APU66KC	MAPT_1	XM_019944330.1	X1	59	397
		APU66KC	MAPT_1	XM_019944331.1	X2	59	397
		APU66KC	MAPT_1	XM_019944332.1	X3	59	310
		APU66KC	MAPT_1	XM_019944333.1	X4	59	397
		APU66KC	MAPT_1	XM_019944334.1	X5	59	397
		APU66KC	MAPT_1	XM_019944335.1	X6	59	310
		APU66KC	MAPT_1	XM_019944336.1	X7	59	397
		APU66KC	MAPT_1	XM_019944337.1	X8	59	310
		APU66KC	MAPT_1	XM_019944338.1	X9	59	110
		APU66KC	MAPT_1	XM_019944339.1	X10	59	110
PSEN1	Presenilin 1	APYMNA3	PSEN1_2	XM_019921707.1	X1	67	1855
		APYMNA3	PSEN1_2	XM_019921714.1	X2	67	1843
PSEN2	Presenilin 2	AP2XCFX	PSEN2_2	XM_019920287.1		55	1812
TARDBP	TAR DNA binding protein	AP7DT6P	TARDBP_1	XM_019930884.1	X1	67	822
		AP7DT6P	TARDBP_1	XM_019930886.1	X3	67	801
RPS9	Ribosomal protein S9	AP324ZV	RPS9_1	EU638309.1	partial cds	55	67
		AP324ZV	RPS9_1	XM_019927380.1	X1	55	207
		AP324ZV	RPS9_1	XM_004332149.2	X2	55	124
		AP324ZV	RPS9_1	XM_019927381.1	X3	55	329
